# DOES NEGATIVE AFFECT MODERATE THE RELATIONSHIP BETWEEN SUBJECTIVE COGNITIVE CONCERNS AND OBJECTIVE FUNCTIONING?

**DOI:** 10.1093/geroni/igac059.2269

**Published:** 2022-12-20

**Authors:** Bradley Dixon, Kylie Kadey, John L Woodard

**Affiliations:** Wayne State University, Detroit, Michigan, United States; Wayne State University, Detroit, Michigan, United States; Wayne State University, Detroit, Michigan, United States

## Abstract

The onset of Alzheimer’s disease can be insidious, slowly affecting an individual’s cognitive abilities. Previous research demonstrated that informant-reported cognitive decline was associated with significantly worse baseline and longitudinal cognitive performance than was a participant’s subjective perceptions of decline. What remains unclear is how negative affect (i.e., depressive symptoms) could moderate the relation between objective cognitive performance and subjectively perceived cognitive concerns (participant vs. informant). Using the National Alzheimer’s Coordinating Center (NACC) database, we performed moderated multiple regressions to test whether Geriatric Depression Scale (GDS) score showed different relationships with cognitive measures (animal/vegetable fluency, digit span, Boston Naming Test, digit-symbol coding (DSC), Wechsler Memory Scale Logical Memory, Trail-Making Test, and Mini-Mental State Examination) for participant- and informant-reported cognitive decline (yes/no). Participants were aged ≥65 years and were cognitively healthy at baseline. Informants lived with the participant or visited the participant weekly (N = 9,354). Participant-reported cognitive concern interacted significantly (p<.05) with negative affect only for animal fluency while informant-reported cognitive concern interacted significantly with DSC. Depressive symptoms were associated more strongly with cognitive performance for participants who did not report a subjective cognitive decline compared to those who did report a subjective cognitive decline. Participant age showed significant negative relationships with all measures while GDS score showed significant negative relationships with all measures except immediate Logical Memory recall, regardless of decline status. In conclusion, negative affect generally did not moderate the relationship between participant- or informant-reported cognitive concerns and objective cognitive functioning except for animal category fluency and DSC.

